# Prognostic Significance of Neutrophil-to-Lymphocyte Ratio for Repeat Cerclage in Women with Prolapsed Membranes

**DOI:** 10.1155/2018/1507398

**Published:** 2018-10-16

**Authors:** Ji Eun Song, Keun Young Lee, Ga Hyun Son

**Affiliations:** Division of Maternal Fetal Medicine, Department of Obstetrics and Gynecology, Hallym University College of Medicine, Seoul, Republic of Korea

## Abstract

**Objective:**

Cerclage is a surgical option for preventing preterm birth (PTB). Repeat cerclage (RC) could prevent impending PTB in women with prolapsed membrane who already had primary cerclage. PTB is associated with a state of inflammation. It has been widely known that neutrophil-to-lymphocyte ratio (NLR) and platelet-to-lymphocyte ratio (PLR) reflect systemic inflammation. We aimed to investigate whether NLR and PLR could be used as reliable markers in predicting pregnancy outcome following RC.

**Methods:**

The study group consisted of 26 patients, who underwent RC resulting from prolapsed membrane after primary cerclage. NLR and PLR at the time of primary cerclage and RC were calculated. ROC curve analysis and multivariate analysis were performed for determining predictive factors. The study group was divided into two groups according to NLR 4.7 at RC: High NLR group was defined as NLR > 4.7, and low NLR group was defined as NLR ≤ 4.7. We compared pregnancy outcomes, such as delivery at gestational age, and rate of delivery < 28 weeks between two groups.

**Results:**

The average gestational age at primary cerclage was 15.7 weeks, and the mean gestational age at RC was 21.0 weeks. NLR at RC was significantly elevated when compared with NLR at primary cerclage (NLR, 4.0 vs. 8.9, p=0.001), but there was no significant difference in terms of PLR (p=0.07). ROC curve showed that there was a significant prediction power of NLR at RC for delivery < 28 weeks (AUC, 0.91; p<0.01). Using NLR cut-off of 4.7, 8 had NLR ≤ 4.7 (low NLR group), whereas 18 had NLR > 4.7 (high NLR group). High NLR group showed worse pregnancy outcome compared to low NLR group: there were significant differences in gestational age at delivery, and neonatal survival rate between two groups (31.5 weeks vs. 25.9 weeks, p=0.02; 100% (8/8) vs. 55.6% (10/18), p=0.03, respectively). Survival analysis demonstrated a lower incidence of delivery < 28 weeks of gestation in low NLR group compared with high NLR group (p<0.01, log-rank test).

**Conclusion:**

NLR might be used as a reliable factor for predicting pregnancy outcome following RC.

## 1. Introduction

Cervical cerclage is a well-known surgical procedure, commonly used during pregnancy for the management of women considered to be at high risk of second-trimester abortion and spontaneous preterm birth (PTB) [1]. Repeat cerclage (RC) is defined as a cerclage which is placed following primary cerclage to prevent PTB [2]. RC could be inserted in women with amniotic membrane prolapse following primary cerclage. We previously reported that RC prolonged pregnancy compared to bed rest group in women with prolapsed membranes after primary cerclage [3]. However, there has been no study on assessing prognostic factors of predicting pregnancy outcome following RC.

Neutrophil-to-lymphocyte ratio (NLR) and platelet-to-lymphocyte ratio (PLR) reflect inflammation severity [4]. NLR and PLR have been proposed as predictive markers for precancerous and cancerous lesions and coronary artery disease which are characterized by inflammatory response [5–7]. The inflammatory process also plays an important role in PTB [3]. Based on these data, we hypothesized that pregnancy outcome after RC may also be related to inflammatory response. We therefore assessed the association between NLR and PLR with pregnancy outcome of RC. We also evaluated the predictive value of these markers on the pregnancy outcome following RC.

## 2. Materials and Methods

Patients who underwent RC at Kangnam Sacred Heart Hospital, Hallym University, between January 2003 and December 2013 were evaluated in this study. 9 of these patients have been reported in a previous study [3]. Inclusion criteria were (i) singleton pregnancy with gestational age between 16 and 25 weeks, (ii) cervical dilation > 1 cm with prolapsed membranes, (iii) intact membranes, (iv) no uterine contraction, (v) absence of vaginal bleeding, and (vi) no fetal abnormalities. Exclusion criteria were multiple gestation, chronic maternal medical diseases, gestational diabetes, preeclampsia, infectious or autoimmune diseases, chorioamnionitis, preterm premature rupture of membrane (PPROM), persistent uterine contraction or alcohol and smoking consumption. Before placing repeat cerclage (a Mc-Donald-type purse string), amniocentesis was performed to reduce the intraamniotic pressure in all patients, and 20cc of amniotic fluid was removed in each patient. The bulging membranes were replaced into the intrauterine cavity with a balloon device. RC was placed using a 5 mm mersilene tape after removing the primary cerclage knot. Intravenous cephalosporin was administered for 3 days postoperatively. In addition, 500mg of oral azithromycin was given routinely. Postoperative patients were treated with absolute bed rest and intravenous ritodrine for postoperative 3 days. The study was approved by the hospital Institutional Review Board. Data were reviewed from medical records retrospectively. A routine biochemistry workup, complete blood count, and fetal ultrasonography were evaluated preoperatively at every time of index cerclage and RC. The routine laboratory measurements of neutrophil, lymphocyte and platelet counts were obtained by systematically search. The neutrophil-to-lymphocyte ratio (NLR) was calculated by dividing the neutrophil concentration by the lymphocyte concentration. The platelet-to-lymphocyte ratio (PLR) was calculated by dividing the platelet concentration by the lymphocyte concentration.

Statistical analysis was performed with the Statistical Package for the Social Sciences (SPSS software version 24.0, Chicago, IL). Data are represented either as number and percent, or means ± standard deviation. Continuous variables were evaluated by Student's *t*-test or Mann–Whitney *U*-test, where appropriate. Chi-square analysis was used for categorical data. Correlations for continuous variables were assessed using either the Pearson or the Spearman test, depending on normal distribution. Cox regressing methodology was used for a stepwise multivariate analysis to determine independent factors affecting the pregnancy outcome following RC. Receiver-operating characteristic (ROC) curves were analyzed to assess the discriminative ability of NLR in RC. Survival curves were compared using Kaplan-Meier analysis and the log-rank test. A *p*-value of <0.05 was considered statistically significant.

## 3. Results

The study cohort consisted of 26 patients. All were Korean. RC was surgically successful in all patients, and there was no intraoperative rupture of bulging membranes or immediate pregnancy loss.  Table 1 summarizes patient characteristics and pregnancy outcomes after RC. The mean gestational age at index cerclage was 15.7 weeks: 50% (13/26) patients underwent history-indicated cerclage (HIC) for previous midtrimester pregnancy losses; 19.2% (5/26) had ultrasound-indicated cerclage (UIC) for short cervical length < 25mm; 30.8% (8/26) underwent physical examination-indicated cerclage (PEIC) for dilated cervix with prolapsed membrane. The mean gestational age at RC was 21.0 weeks. The average size of prolapsed membrane into the vagina was 3.3 cm. The mean gestational age at delivery following RC was 27.6 weeks with 69.3% (18/26) neonatal survival rate. 8 patients (30.7%) ended with immediate neonatal death resulting from extreme prematurity. 65.4% (17/26) delivered prematurely for preterm labor: 9 patients had cesarean section for placenta previa or fetal malposition such as breech presentation or transverse lie; 8 patients had vaginal delivery after removing cerclage knot. 34.6% (9/26) patients delivered for premature rupture of membrane (PROM). There was no cervical laceration or amputation, because patients had immediate treatment accordingly. There was a significant difference between mean NLR at index cerclage and NLR at RC (4.0 ± 0.9 vs. 8.9 ± 6.6, p<0.01). Despite the increase of PLR at RC compared to PLR at index cerclage, there was no statistically significance (173.2 ± 38.2 vs. 194.6 ± 46.5, p=0.07). NLR at RC was a powerful predictor of PTB in both univariate (Odds ratio [OR]=1.9; 95% CI: 1.1-3.3; p=0.01) and multivariate analyses (OR=2.6; 95% CI: 1.1-6.0; p=0.02).

**Figure 1:**
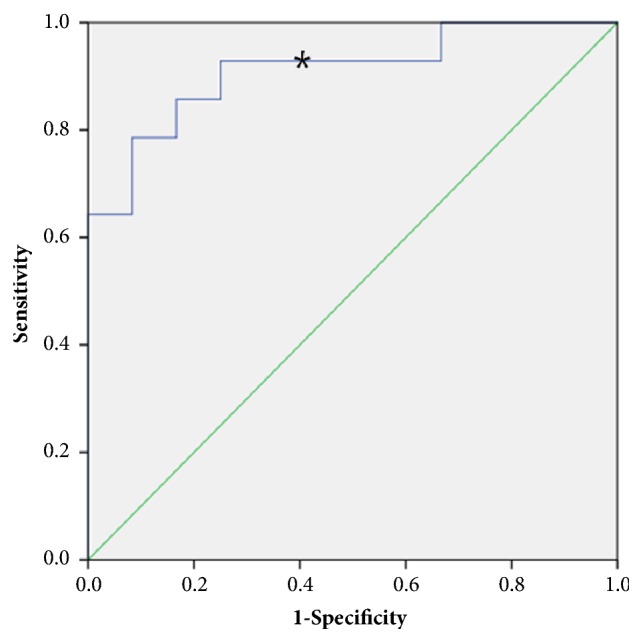
Receiver-operating characteristic (ROC) curve of NLR at RC predicting delivery < 28 weeks of gestation. The areas under curve (AUCs) are 0.91 (p <0.01). *∗* NLR 4.7 with specificity 41.7% and sensitivity 92.9%.

Receiver-operating characteristic (ROC) curve analysis demonstrated that NLR at RC may be a discriminative parameter for predicting delivery < 28 weeks of gestation. The area under curve (AUC) of ROC curve for the prediction of delivery < 28 weeks of gestation by NLR was 0.91 (p<0.01) (Figure 1). Using an NLR cut-off of > 4.7 at RC, the sensitivity and specificity of predicting delivery < 28 weeks of gestation was 92.9 % and 41.7 %, respectively. Using NLR cut-off of > 4.7 at RC, 8 had NLR ≤ 4.7 (low NLR group), whereas 18 had NLR > 4.7 (high NLR group).  Table 2 demonstrates the comparison of pregnancy outcomes according to NLR at RC. There was no significant difference in the diameter of bulging membrane at RC between two groups (3.1cm vs. 3.4cm, p=0.65). There were no statistical differences in average NLR and PLR at prior cerclage between two groups (NLR, 3.6 vs. 4.2, p=0.15; PLR, 159.2 vs. 179.4, p=0.22). However, the mean NLR at RC differs significantly between two groups (3.6 vs. 11.2, p=0.004). There was no statistical difference in PLR at RC, although the average PLR at RC was increased in high NLR group (174.3 vs. 203.7, p=0.14). High NLR group showed worse pregnancy outcome compared to low NLR group. There were significant differences in gestational age at delivery, birthweight, and neonatal survival rate between two groups (31.5 weeks vs. 25.9 weeks, p=0.02; 2095.0g vs. 1081.1g, p=0.01; 100%(8/8) vs. 55.6%(10/18), p=0.03, respectively).

**Figure 2:**
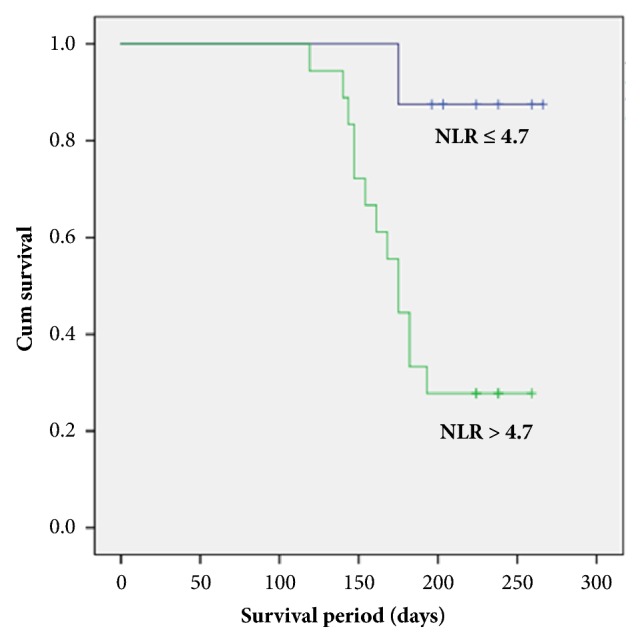
Kaplan-Meier survival curve for delivery < 28weeks of gestation with NLR ≤ 4.7 and > 4.7.

There were 14 deliveries < 28 weeks of gestation during the course of the study. Kaplan-Meier survival curves showed a lower incidence of delivery < 28 weeks in the NLR ≤ 4.7 group (12.5 %) compared with the NLR > 4.7 group (72.2 %) (p< 0.01, log-rank test) (Figure 2). This shows that the rate of delivery < 28 weeks of gestation was significantly worse in patients with NLR > 4.7.

## 4. Discussion

The principal finding of our study is that NLR is a valuable predictor of pregnancy outcome following RC. NLR at the time of RC was significantly elevated compared to that of primary cerclage. Moreover, ROC curve and multivariate analysis demonstrated that NLR was a discriminative parameter for predicting delivery < 28 weeks of gestation. We found that patients with elevated NLR showed subsequent worse pregnancy outcome: NLR > 4.7 was associated with earlier delivery, lower birthweight, and poorer neonatal survival rate. These findings may reflect the hidden connection between NLR and immunological inflammatory process in women with RC.

NLR and PLR are simple and widely available markers for systemic inflammation, which can be easily obtained from complete blood counts (CBC) in peripheral blood [4]. NLR is currently considered to be a prognostic factor in oncologic, cardiac disease, and peripheral vascular surgery [8, 9]. PLR is reported to be associated with breast, pancreatic, and colorectal cancers [4, 5]. In obstetrics and gynecology, NLR and PLR has been reported to be correlated with ovarian cancer [10, 11] Another study suggested that NLR is associated with hormonal status in polycystic ovarian syndrome [12]. Recent study reported that NLR could identify PTB in women with spontaneous, late preterm labor [13]. In the study, NLR cut-off of 6.2 was used, and high NLR (> 6.2) at admission could predict PTB in women with threatened preterm labor between 34 and 37 weeks of gestation [13]. Our current study also showed that elevated NLR predicted PTB < 28 weeks of gestation in RC. However, PLR did not have significant prognostic value. These findings support the use of NLR as a useful tool for predicting pregnancy outcome, and it may enable obstetricians to counsel patients with high risk pregnancy.

In the previous published study, we report that RC prolongs pregnancy in women with prolapsed membrane compared to bed rest group [3]. There is no uniform agreement on the most appropriate therapeutic methods for managing short cervix or prolapsed membrane after a prior failing cerclage attempt. Some have suggested that RC did not improve pregnancy outcome in women with sonographically short cervix after index cerclage [14–16]. However, our previous study differs from those papers. We performed RC in women with bulging membrane after a prior cerclage. Prolapsed membrane leads to previable PTB, when no further intervention is performed [17]. Our previous study showed that RC effectively enhanced pregnancy outcome in those circumstances [3]. To the best of our knowledge, our current study is the first to show that NLR is a valuable factor for predicting pregnancy outcome following RC in women with prolapsed membrane after primary cerclage. Our finding adds further support to the proposal that a systemic inflammatory reaction is associated with subsequent poor outcome in RC.

There are some limitations. First, it was a retrospective and single-institution investigation with a relatively small sample size. Second, we did not correlate NLR with other possible inflammatory markers in amniotic fluid or umbilical cord samples. Further studies on the correlation of amniotic fluid biomarkers and NLR are warranted. Lastly, future studies on the uniform agreement of cut-off value of NLR are needed. We suggested NLR 4.7 in this study, but other researchers reported NLR 6.2 as the risk factor for preterm birth [13]. Despite these limitations, this study showed that NLR could independently predict pregnancy outcome in women undergoing RC. NLR is an inexpensive and easily calculated marker, which may provide additional prediction of postoperative pregnancy prognosis. Further prospective study is needed to enlighten the underlying mechanisms and other valuable parameters of RC using various samples.

## Figures and Tables

**Table 1:** Patient characteristics in repeat cerclage (n=26).

Variables	Values
Age (years)	32.5 (± 3.3)
BMI (kg/m^2^)	25.2 (± 4.0)
Primigravida	10/26 (38.4%)
Type of prior cerclage	
History indicated cerclage (HIC)	13/26 (50%)
Ultrasound-indicated cerclage (UIC)	5/26 (19.2%)
Physical examination-indicated cerclage (PEIC)	8/26 (30.8%)
GA at prior cerclage (weeks)	15.7 (± 3.1)
GA at repeat cerclage (weeks)	21.0 (± 2.3)
NLR at prior cerclage	4.0 (± 0.9)
PLR at prior cerclage	173.2(± 38.2)
NLR at repeat cerclage	8.9 (± 6.6)
PLR at repeat cerclage	231.2(± 125.2)
Bulging membrane at the time of repeat cerclage (cm)	3.3 (± 1.6)
GA at delivery (weeks)	27.6 (± 5.8)
Latency between repeat cerclage and delivery (days)	46.5 (± 39.9)
Birthweight (g)	1393.0 (± 1037.2)
Neonatal survival	18/26 (69.2 %)

Note: NLR, neutrophil/lymphocyte ratio; PLR, platetlet/lymphocyte ratio; GA, gestational age;

Values are n, mean (± standard deviation) or n/N (%).

**Table 2:** Pregnancy outcome according to NLR at repeat cerclage.

	NLR ≤ 4.7 (n=8)	NLR > 4.7 (n=18)	*p* value
Age (years)	33.7 (± 3.9)	32.0 (± 2.9)	0.23
BMI (kg/m^2^)	27.5 (± 4.5)	24.2 (± 3.5)	0.05
Primigravida	2/8 (25%)	8/18 (44.4%)	0.42
NLR at prior cerclage	3.6 (± 0.5)	4.2 (± 1.0)	0.15
PLR at prior cerclage	159.2 (± 18.5)	179.4 (± 43.3)	0.22
NLR at repeat cerclage	3.6 (± 0.8)	11.2 (± 6.7)	0.004*∗*
PLR at repeat cerclage	174.3 (± 26.2)	203.7 (± 51.2)	0.14
Bulging membrane at repeat cerclage (cm)	3.1 (± 1.2)	3.4 (± 1.8)	0.65
GA at prior cerclage (weeks)	15.7 (± 2.8)	15.7 (±3.3)	0.99
GA at repeat cerclage (weeks)	20.2 (± 1.1)	21.3 (± 2.7)	0.27
GA at delivery (weeks)	31.5 (± 4.5)	25.9 (± 5.6)	0.02*∗*
Birthweight (g)	2095.0 (± 1063.0)	1081.1 (± 884.2)	0.01*∗*
Neonatal survival	8/8 (100%)	10/18 (55.6%)	0.03*∗*
Delivery < 28 weeks of gestation	1/18 (12.5%)	13/18 (72.2%)	<0.01*∗*

Note: NLR, neutrophil/lymphocyte ratio; PLR, platelet/lymphocyte ratio; GA, gestational age

Values are n, mean (± standard deviation) or n/N (%).

*∗* Statistically significant.

## Data Availability

The data of our manuscript would be provided when requested.
